# Consider the hubris syndrome for inclusion in our classification systems

**DOI:** 10.1017/S0033291723002672

**Published:** 2023-10

**Authors:** Jean-Paul Selten

**Affiliations:** School for Mental Health and Neuroscience, University of Maastricht, Maastricht, the Netherlands

**Keywords:** antisocial personality disorder, diagnostic validity, hubris, narcissistic personality disorder, personality change, psychopathy

## Abstract

Successful leaders are at risk of developing exaggerated pride, contempt for others, and a diminished sense of reality. The ancient Greeks feared this syndrome and called it hubris. Although certain contemporaneous leaders show signs of hubris and pose a great danger, the hubris syndrome does not yet figure in our classification systems. The purpose of this paper is to examine several aspects of its validity, including clinical description, laboratory study, and exclusion of other disorders. Firstly, a substantial body of evidence indicates that the hubris syndrome may develop after a person has held substantial power for a considerable amount of time. Thus, the syndrome differs from a personality disorder with its characteristic onset in late adolescence or early adulthood. It is proposed, therefore, that the syndrome is a non-organic personality change after gaining substantial power or achieving overwhelming success, characterized by the emergence or marked increase of pathological personality traits within the domains of dissociality and disinhibition. Within the domain of dissociality, grandiosity is an obligatory trait. Secondly, with reference to laboratory study, recent evidence suggests that machine learning algorithms have the ability to differentiate hubristic from non-hubristic speech patterns. Thirdly, the exclusion of other disorders is difficult, because individuals with the hubris syndrome do not collaborate in any investigation. Some suggestions are made to overcome this problem. In conclusion, there is sufficient reason to further examine the validity of the hubris syndrome and to consider it for inclusion in our classification systems.

Successful leaders are at risk of developing exaggerated pride, contempt for others, and a diminished sense of reality. The ancient Greeks feared this development and called it hubris. History offers many examples. A notorious case from military history is Napoleon's campaign into Russia and his tragic march home, which cost the lives of 400 000 to 500 000 soldiers. Another example is Mao Zedong who, in 1958, received Soviet-leader Khrushchev by the side of a swimming pool, clad only in a bathrobe and slippers. Without any knowledge of agriculture or metallurgy, he imposed a project for a quick industrialization of China (‘Great Leap Forward’, 1958–1962), which caused a famine and 15–55 million casualties (https://en.wikipedia.org/wiki/Great_Leap_Forward; Dikötter, 2017; Li, 1994).

The leadership literature recognizes the hubris syndrome as a category of destructive leadership, which poses a danger not only to the nation but also to companies (Garrard, 2018; Petit & Bollaert, 2012; Sadler-Smith, Akstinaite, Robinson, & Wray, 2017). Since hubristic leaders are contemptuous to the advice of others, over-ambitious, and reckless in strategic choices, the early identification and prevention of CEO hubris are important aims of research. Reports of hubristic behavior also concern leaders of universities and other prestigious organizations. David Owen, a neurologist and a former foreign secretary of the United Kingdom (1977–1979), described the hubris syndrome in several modern leaders and proposed diagnostic criteria (Owen & Davidson, 2009). Apart from a positive response from Russell (2011), this initiative has not yet led to lively discussion among mental health professionals.

Is there sufficient evidence to warrant further study of the hubris syndrome for a possible inclusion in a next edition of our classification systems? Critics will argue that we should not be too quick to label extremes of behavior as pathological, but the thoughts and actions of certain contemporaneous leaders are very abnormal and dangerous. In order to avoid possible libel charges, I do not mention names of living persons. The recognition of the syndrome as a separate diagnostic entity would stimulate discussion as to whether a leader meets the criteria and may contribute to the implementation of measures to prevent its development. In this attempt to answer the question, I will take as guide Robins and Guze method for establishing the validity of a diagnostic entity, consisting of five phases: clinical description, laboratory study, exclusion of other disorders, follow-up, and family study (Robins & Guze, 1970).

As for the first phase, Owen and Davidson (2009) rightly argued that a diagnosis of hubris syndrome can only be made in an individual who holds substantial power for a certain amount of time and who develops the characteristic symptoms after the acquisition of this power. Thus, they consider the syndrome as an acquired personality disorder that appears at an older age than the personality disorders, which have their onset in adolescence or early adulthood. In a second step, they proposed 14 criteria for the syndrome, 9 of which overlap with those for the narcissistic, antisocial, or histrionic personality disorder listed in the Diagnostic and Statistical Manual of Mental Disorders, 4th edition (DSM-IV) (American Psychiatric Association, 1994) and 5 that they consider to be unique to the hubris syndrome, unique in the sense that they have not been classified elsewhere: (i) identification with the nation or organization to the extent that the individual regards his/her outlook and interests as identical; (ii) tendency to speak in the third person or use the royal ‘we’; (iii) unshakable belief to be vindicated in court; (iv) restlessness, recklessness, and impulsiveness; (v) tendency to allow their ‘broad vision’, about the moral rectitude of a proposed course, to obviate the need to consider practicality, cost, or outcomes. Owen and Davidson suggested that an individual should meet at least 3 of the 14 features and that at least one of them should figure among the five symptoms identified as unique to the hubris syndrome.

Although this is a very good description of the hubris syndrome, some criteria are rather complex blends of symptoms. Moreover, the classification of personality disorders is moving away from a categorical to a dimensional approach. While the Diagnostic and Statistical Manual of Mental Disorders, 5th edition (DSM-5) retained the categorical system and mentioned the dimensional approach as a possible alternative (American Psychiatric Association, 2013), the International Classification of Diseases, 11th Revision, switched to the dimensional approach in 2022 (https://icd.who.int/en). Briefly, the ICD-11 system acknowledges the ‘big 5’ dimensions in personality (openness, consciousness, extraversion, agreeableness, and neuroticism) and asks the clinician, after a categorical yes/no decision about the presence of a personality disorder, to describe the disorder using one or more of five personality trait domains that connect to these dimensions. These trait domains are negative affectivity, detachment, dissociality, disinhibition, and anankastia. Each domain comprises a spectrum of personality traits or facets that tend to occur together, but to preserve parsimony only the domain level is used for clinical description (e.g. Bach *et al*., 2022; Oltmans, 2021). Obviously, dissociality and disinhibition are the domains relevant for the hubris syndrome. The core features of these dimensions are disregard for the rights and feelings of others (dissociality) and the tendency to act rashly without consideration of potential negative consequences (disinhibition). Common manifestations of dissociality include self-centeredness (e.g. sense of entitlement, expectation of others' admiration, attention-seeking behaviors) and lack of empathy (e.g. callousness in response to others' suffering and ruthlessness in obtaining one's goals). Common signs of disinhibition are irresponsibility, impulsivity, distractibility, recklessness, or lack of planning (https://icd.who.int/en). All things considered, it is proposed here that the hubris syndrome is a *non-organic personality change* after gaining substantial power or achieving overwhelming success, characterized by the *emergence or marked increase* of pathological traits within the domains of dissociality and disinhibition. Moreover, the individual in question should manifest, within the domain of dissociality, the pathological trait of grandiosity. The choice of this criterion is supported by the fact that 12 of 14 criteria formulated by Owen and Davidson refer to grandiosity (only criteria 11 and 12 do not). Since arrogant individuals do not cause much harm when they are not disinhibited, the emergence or marked increase of at least one of the following traits within the domain of disinhibition is also required for a diagnosis: irresponsibility, impulsivity, recklessness, or lack of planning. (Not distractibility, because it does little damage to others.) The details of this proposal are given in Table 1. It is true, to date there are no diagnostic categories for non-organic personality changes, but it is likely that more such personality changes exist. One could think, for instance of the low-level of paranoia in many prisoners.

**Table 1.** Hubris syndrome: a proposal for diagnostic criteria

A syndrome characterized by a personality disturbance that represents a change from the individual's previous characteristic personality pattern and has been present for at least 3 months. The personality disturbance meets the criteria for a personality disorder, except the onset in late adolescence or early adulthood. The personality disturbance has developed after achieving overwhelming success or gaining considerable power. The personality disturbance is characterized by the emergence or marked increase of pathological traits within the domains of dissociality and disinhibition. The pathological trait(s) within the domain of dissociality include grandiosity: believing that one is superior to others; self-centeredness; feelings of entitlement; condescension toward others.^a^ The pathological traits within the domain of disinhibition are one or more of the following: impulsivity, irresponsibility, recklessness, and lack of planning. The disturbance is not accounted for by another mental or behavioral disorder and is not a pathophysiological consequence of a health condition not classified under mental and behavioral disorders.

a This description of grandiosity has been derived in part from ‘Alternative DSM-5 model for personality disorders’, in: American Psychiatric Association (2013), *Diagnostic and Statistical Manual of Mental Disorders* (5th ed.). Washington, DC: APA, pp. 761–781.

*Note:* The presence of a personality disorder with the usual onset in late adolescence or early adulthood does not preclude an additional diagnosis of the hubris syndrome.

With reference to the second phase of validation (laboratory study), research of speech patterns of hubristic and non-hubristic leaders supported the second ‘unique’ criterion for the hubris syndrome, a tendency to speak in the ‘royal we’. When the researchers counted the frequency of the first person plural pronouns (‘we’, ‘us’, and ‘our’) and their singular equivalents (‘I’, ‘me’, and ‘my’), they found evidence of an increased We-to-I ratio in the affected leaders (Garrard, Rentoumi, Lambert, & Owen, 2014; Magyari, Pléh, & Forgács, 2022). The results of a recent study suggest that machine learning algorithms have the ability to identify automatically hubristic *v.* non-hubristic speech patterns (Akstinaite, Garrard, & Sadler-Smith, 2022). This is an interesting area for further study.

The third phase, which concerns the exclusion of other disorders, is the most important challenge. It is not so hard to distinguish the hubris syndrome from an antisocial personality disorder, mania, hypomania, or conditions brought about by the use of amphetamines or cocaine, but it is more difficult to separate it from a narcissistic personality disorder. There are two important points of consideration here. First, it is likely that most individuals who develop the hubris syndrome functioned very well before they acquired power. It is after all difficult to win elections or to make a brilliant career when one exhibits exaggerated pride, overwhelming self-confidence, and contempt for others. Second, the onset of a personality disorder is in late adolescence or early adulthood, while that of the hubris syndrome is generally much later. In order to test this idea, we need to examine whether the hubris syndrome can indeed be distinguished from pre-existing personality disorders.

This brings us straight to the problem that individuals with the hubris syndrome are highly unlikely to collaborate in any investigation. The same difficulty arises, of course, if one tries to assess the interrater reliability of the diagnosis: face-to-face interviews attended by several observers, the usual way to assess interrater reliability, are not feasible. This is probably one of the reasons why mental health professionals have so far shied away from the syndrome. It is nonetheless important to solve this difficulty, for a syndrome does not deserve a scientific status if experts do not agree on its presence or absence. We need to reach agreement on a set of clear diagnostic criteria and should not be afraid to use creative methods to examine their reliability and validity. I see at least two possibilities for doing this: (i) a prospective study with very successful young politicians, businessmen, or businesswomen who agree to be followed over time; (ii) a working group of experts that follows leaders of government who have been in office for more than 8 or 10 years. (Since the term for most governments is 4–5 years, this means that they have obtained a third term.) An increasing body of evidence suggests that they are at ultra-high risk of developing the syndrome. The results of these studies may lead to a revision of the proposed criteria.

With regard to the fourth phase of validation (follow-up), I am not aware of any study that examined the course of the syndrome systematically. Anecdotal evidence suggests that the lack of self-criticism is often persistent.

As for the fifth phase, the main purpose of family studies is to examine whether there is a genetic contribution to etiology. However, since the acquisition of great power is a rare event that happens almost always to one member of the family, such studies are not very useful. One could argue that this is not true for royal families like the Romanovs or Habsburg, but the members of these families did not consider the acquisition of power as an achievement to be proud of, but as a natural event.

In sum, there is sufficient reason to consider the hubris syndrome for inclusion in our classification systems. The recognition of the hubris syndrome as a valid diagnostic category and a danger to mankind will constitute an important step toward prevention. The United States of America did well when they ruled that a president can be re-elected only once. Other countries should follow this example and the international community should issue a world-wide ban of leaders who hold power for more than 8 years.

## Funding statement

This research received no specific grant from any funding agency, commercial, or not-for-profit sectors.
